# Molecular modeling and expression analysis of a *MADS*-*box* cDNA from mango (*Mangifera indica* L.)

**DOI:** 10.1007/s13205-013-0162-0

**Published:** 2013-08-20

**Authors:** Magda A. Pacheco-Sánchez, Carmen A. Contreras-Vergara, Eduardo Hernandez-Navarro, Gloria Yepiz-Plascencia, Miguel A. Martínez-Téllez, Sergio Casas-Flores, Aldo A. Arvizu-Flores, Maria A. Islas-Osuna

**Affiliations:** 1 Plant Molecular Biology Lab, Centro de Investigación en Alimentación y Desarrollo, A.C., Carretera a la Victoria Km 0.6, Apartado Postal 1735, 83304 Hermosillo, Sonora Mexico; 2 Departamento de Ciencias Químico Biológicas, Universidad de Sonora, Blvd. Luis Encinas y Blvd. Rosales S/N, 83000 Hermosillo, Sonora Mexico; 3 División de Biología Molecular, IPICYT, Camino a la Presa San José No. 2055, Lomas 4a sección, 78216 San Luis Potosí, Mexico

**Keywords:** *Mangifera indica* L., Gene expression, MADS-box, Molecular modeling, Transcription factor

## Abstract

*MADS*-*box* genes are a large family of transcription factors initially discovered for their role during development of flowers and fruits. The MADS-box transcription factors from animals have been studied by X-ray protein crystallography but those from plants remain to be studied. In this work, a *MADS*-*box* cDNA from mango encoding a protein of 254 residues was obtained and compared. Based on phylogenetic analysis, it is proposed that the MADS-box transcription factor expressed in mango fruit (MiMADS1) belongs to the SEP clade of MADS-box proteins. *MiMADS1* mRNA steady-state levels did not changed during mango fruit development and were up-regulated, when mango fruits reached physiological maturity as assessed by qRT-PCR. Thus, MiMADS1 could have a role during development and ripening of this fruit. The theoretical structural model of MiMADS1 showed the DNA-binding domain folding bound to a double-stranded DNA. Therefore, MiMADS1 is an interesting model for understanding DNA-binding for transcriptional regulation.

## Introduction

Gene expression associated to specific inductive events on fruit development is poorly characterized in fruits of commercial importance. In a plant cell, the temporal and spatial organization of these events is mediated by transcription factors required for gene expression. The MADS-box proteins comprise one of the largest groups of plant transcription factors with diverse functions during important events of plant development. *MADS*-*box* genes represent a highly conserved gene family encoding transcription factors in plants, which contain a conserved sequence called MADS-box as an acronym of the first four identified members of the family (MADS: MCM-AGAMOUS-DEFICIENS-SRF) (Liu et al. 2009), and have regulatory functions in flower and fruit development (Theißen and Saedler 2001). More recently, studies of ripening-inhibited spontaneous tomato mutants have shown that *MADS*-*box* genes play a major role in the molecular regulation of development in tomato fruit (Elitzur et al. 2010), aside of their role in organ and flower development initially described in *Arabidopsis* (Weigel and Meyerowitz 1994).

The function of MADS-box protein family on regulation of gene expression have been extensively studied, mainly the floral organ identity in *Arabidopsis* (Ito et al. 2008). Using information of mutants in *Arabidopsis thaliana* and *Antirrhinum majus* in which some identity of floral organs is changed, the ABC model is the most simple to explain how identity arises during development (Theißen and Saedler 2001). Tomato MADS-RIN protein regulates tissue differentiation acting as a transcription factor. Development of fleshy fruits such as tomato involves cell division and expansion of the ovary tissues. Recent studies in *A. thaliana* and tomato have been important to elucidate the function of MADS-box transcription factors on ethylene biosynthesis, perception and signaling pathways. Mango (*Mangifera indica* L.) is one of the most important tropical fruits and it could serve as a model to study development and ripening of this fruit.

The *MADS*-*box* gene family encodes transcription factors present in a variety of organisms in diverse kingdoms. An exclusive family for plant MADS proteins belong to the MICK-type that includes a MADS (M), intervening (I), keratin like (K) and C-terminal (C) domains (Kaufmann et al. 2005). MADS-box proteins have in common a highly conserved DNA-binding domain located at the N-terminal, this sequence of approximately 60 amino acids is common to all MADS type proteins. The MADS-box domain binds to a conserved DNA motif know as CArG box [CC(A/T)_6_GG]. In terms of structure, the DNA-binding domain of MEF2A has been determined by X-ray diffraction for the myocyte enhancer factor 2 (MEF2), which is involved in muscle development (Han et al. 2005; Wu et al. 2010), and also by NMR (Huang et al. 2000). In this work, a *MADS*-*box* cDNA was cloned, its deduced amino acid sequence was compared and modeled bound to DNA, and *MiMADS1* mRNA levels were assayed at different mango fruit developmental stages.

## Materials and methods

### cDNA isolation and analysis of MiMADS1 amino acid sequence

Total RNA from mango pulp was isolated as described elsewhere (Lopez-Gomez and Gomez-Lim 1992), and purified by Oligotex Direct mRNA Mini Kit (QIAGEN). The cDNA was synthesized with the SMART cDNA library construction kit (Clontech) following manufacturer’s recommendations. Three independent *MADS*-*box* cDNA clones were sequenced thoroughly at the Genomic Analysis and Technology Core at the University of Arizona (Tucson, AZ, USA). These three clones were piled up to form a contiguous unambiguous clone and its identity was obtained after a BLASTX search. The cDNA contig was named MiMADS1 and deposited in GenBank. The deduced amino acid sequence was obtained and compared to other MADS-box transcription factors with CLUSTAL W using the T-Coffee server (Notredame et al. 2000).

### MiMADS1 phylogenetic analysis

A phylogenetic analysis was done using the Maximum Likehood algorithm. The bootstrap consensus tree inferred from 1,000 replicates was taken to represent the evolutionary history of the analyzed taxa (Felsenstein 1985). The analyses were conducted with MEGA5 software (Tamura et al. 2011).

### Molecular modeling

A molecular model of MiMADS1 was obtained by homology modeling using the MOE v2012.10 software. The crystallographic structure used, as template was the MEF2A bound to DNA deposited with the PDB code 1EGW (Santelli and Richmond 2000). A multiple sequence alignment was done previously to match the conserved residues from MiMADS1 and MEF2A N-terminal domain. Then, 25 intermediate models were constructed under the CHARMM27 force-field and DNA bounded from the template coordinates was included for induced fit simulation on MiMADS1 model. The final model was further refined under the default parameters of MOE. Figures from the molecular model were done with MOE software.

### *MiMADS1* gene expression

Mango (*Mangifera indica* L.) fruits cv. Keitt were hand harvested during four different developmental stages in a commercial packinghouse at El Porvenir, Sinaloa, México (25°55′56.83″N, 109°5′40.11″W) and transported to the laboratory. Fruits were selected and washed with chlorinated water (200 ppm sodium hypochlorite), numbered and weighed. Fruits were taken and the peel was removed and cut to obtain small pieces of the pulp and were immediately frozen with liquid nitrogen.

Total RNA extraction and cDNA synthesis was done using frozen mango mesocarp tissue as described (1992). The extracted RNA was cleaned with RNAse free DNase I (Roche). RNA quantity was estimated at 260 nm using a NanoDrop ND-1000 ultraviolet–visible spectrophotometer (NanoDrop Technologies Inc., Wilmington, DE, USA). The integrity of total RNA was evaluated by formaldehyde-agarose gel electrophoresis (Sambrook and David 2001). The cDNAs were synthesized from 5 μg of total RNA using a cDNA synthesis kit (Invitrogen) that was prepared from the pool of five experimental units.

Relative expression of *MiMADS1* was evaluated by qRT-PCR and the *18S* rRNA gene was used for data normalization. Real-time PCR was realized in a StepOne Real-Time PCR (Life Technology, CA) using iQSYBR Green Supermix (BIO-RAD). For each gene, a standard curve was generated using cDNA serial dilutions and PCR efficiencies were calculated from slope according to the manufacturer’s instructions. The specific primers for *MiMADS1* were MiMADS1-Fw: 5′-ATGGGATCAAGCTGGAGTAC-3′ and MiMADS1-Rv: 5′TCAAAGCATCCATCCTGG-3′, primers for *18S* rRNA were Mi18S-Fw: 5′-GGTGACGGAGAATTAGGGTTC-3′ and Mi18S-Rv: 5′-CCGTGTCAGGATTGGGTAAT-3′. qRT-PCR reactions were carried out using 4 ng of cDNA under the following conditions: 94 °C for 30 s as denaturation step, followed for 35 cycles of: 94 °C for 30 s, 60 °C for 30 s, 72 °C for 1 min, and 72 °C for 5 min as final extension. The data were analyzed using the 2^−ΔΔCt^ method for calculation of relative changes in gene expression (Livak and Schmittgen 2001) where the fold change was calculated as follows:

$$\mathrm{Fold}\;\mathrm{change}=\;2^{-\Delta \Delta \mathrm{Ct}}=[({\mathrm{Ct}}_{\mathrm{Target}}-{\mathrm{Ct}}_{18S}){\mathrm{Time}}_{x}]-[({\mathrm{Ct}}_{\mathrm{Target}}-{\mathrm{Ct}}_{18S}){\mathrm{Time}}_{0})].$$

Thus, the relative expression levels reported here were calibrated and normalized to the constitutive gene *18S* rRNA.

### Statistical analysis

The data were analyzed as a completely randomized design with three replicates using the GLM procedure of NCSS 2007. Comparison of mean was performed using the multiple range test of Tukey–Kramer with a significance level of 0.05.

## Results and discussion

### cDNA and deduced amino acid sequence of MiMADS1

**Fig. 1:**
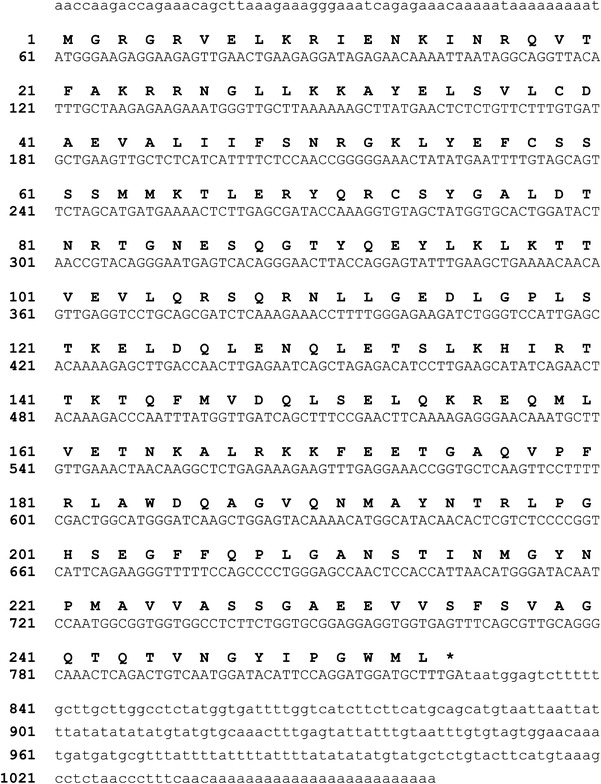
The cDNA and deduced amino acid sequence of MiMADS1. The lower case letters indicate the 5′-untranslated region and the 3′-untranslated region

**Fig. 2:**
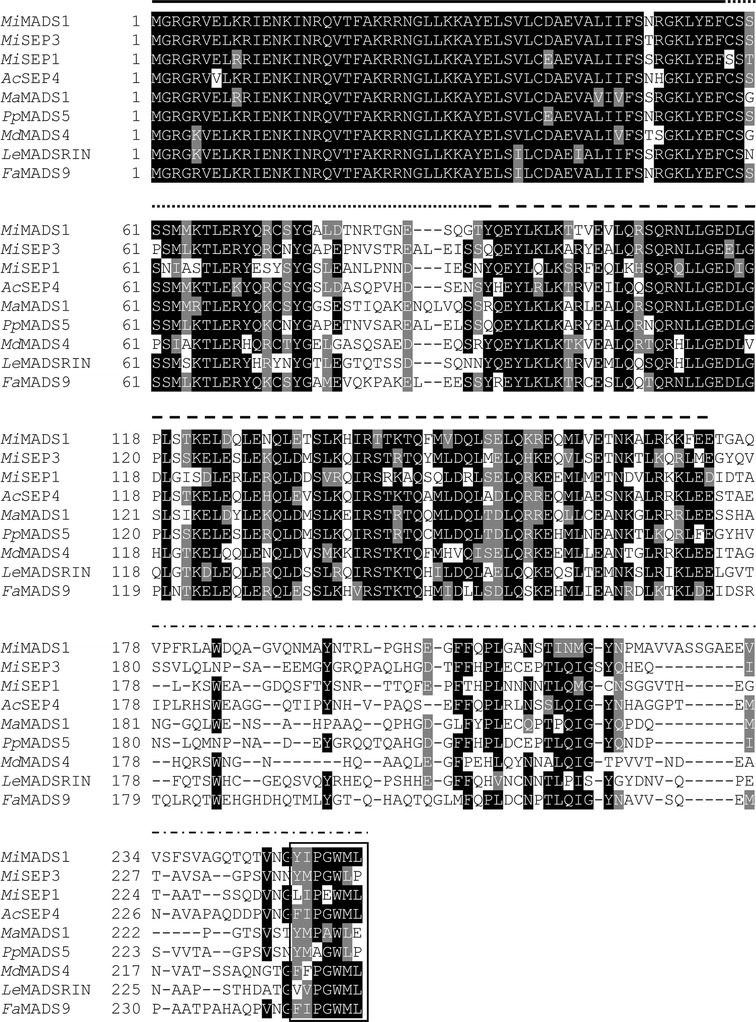
Sequence alignment of selected MADS-box proteins: MiMADS (KF214778) this work, MiSEP3 (AEO45959) (mango), MiSEP1 (ADX97328) (mango), AcSEP4 (ADU15479) (kiwifruit), MaSEP3MADS1 (ACJ64679) (banana), PpMADS5 (AAZ16241) (peach), MdSEP4MADS4 (AAD51423) (apple), LeMADSRINSEP4 (NP_001234670) (tomato), FaMADS9 (XP_004304204) (woodland strawberry)

The complete sequence of the *MiMADS1* cDNA (GenBank KF214778) was 1,062 bp with a 62 bp 5′-untranslated region, a 765 bp Open Reading Frame including the initial methionine and the stop codon and a 3′-untranslated region of 195 nt and a 24 nt poly-A^+^ tail (Fig. 1). The encoded protein of 254 residues has a theoretical pI of 9.1 and a molecular weight of 29.7 kDa. Multiple amino acid sequence alignment showed that MiMADS1 is similar to MADS-box proteins from other fruits (Fig. 2) for example it is 70 % identical to PpMADS5 from peach (*Prunus persica*, AAZ16241) (Xu et al. 2008) and 68 % identical to AcSEP4 from kiwifruit (*Actinidia chinensis*, ADU15479) (Varkonyi-Gasic et al. 2011).

MiMADS1 has a MADS-box domain of 57 residues based on sequence comparison to other MADS-box transcription factors. The I domain (linker region) of MiMADS1 has a length of 33 residues, which is similar in length to the MICK protein QUAMOSA (SQUA) that has an I domain of 35 residues (Henschel et al. 2002). Also, the conserved K domain of MiMADS1 consists of three subdomains that form alpha helixes with hydrophobic amino acids (Kaufmann et al. 2005). The structure of the K domain is important for protein–protein interactions since point mutations made in this domain have shown its importance. Furthermore, the coiled-coil structure of K domain is important for protein–protein interaction. The linker region is variable and located between the MADS- and K-domains, which shows sequence and structural similarity to the coiled-coil domain of keratin, likely forms amphipathic α-helices. Both the I and K-domains, are implicated in determining protein–protein dimerization specificity (Cseke and Podila 2004). The C region is not required for dimerization but it is needed for the formation of ternary complexes (Aswath and Kim 2005). Moreover, the C domain, located at the C terminus is the domain that has the capacity of transcriptional activation (Immink et al. 2009). In fruits like apple, grapes and peach, expression of *MADS*-*box* genes has been observed during early stages of fruit development (Yao et al. 1999; Choudhury et al. 2012). MADS-box proteins can form homo or heterodimers as it has been demonstrated by a large number of in vitro and yeast two-hybrid experiments. Structure of these proteins will also help to elucidate functional properties related to their structure (Smaczniak et al. 2012).

### MiMADS1 phylogenetic analysis

Sequence analysis showed that MiMADS1 protein primary structure is similar to members of the SEP clade. SEP are transcription factors that facilitate the formation of complexes along with other MADS-box proteins and activate the potential of these complexes due to the fact that members of the SEP subfamily have a role as mediators of higher order complex formation (Ito et al. 2008; Smaczniak et al. 2012). The amino acid sequence of MiMADS1 was aligned with other MADS-box proteins and it is more similar to proteins from the SEP and AGL families.

**Fig. 3:**
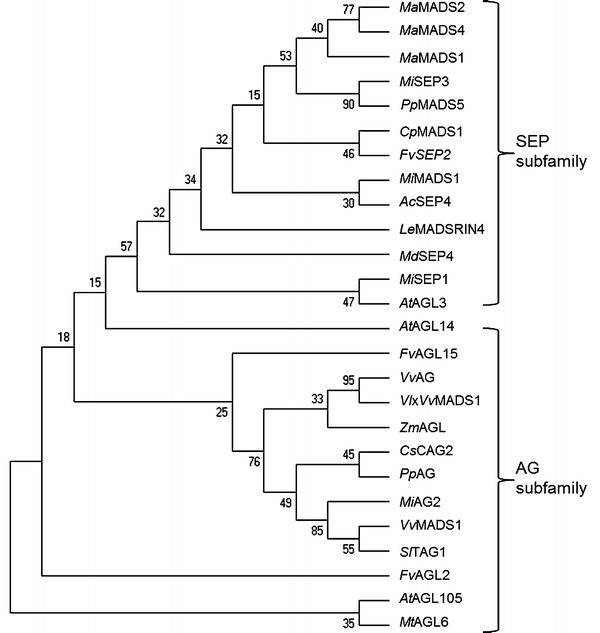
Phylogenetic analysis of MADS proteins by Maximum Likelihood method. The protein sequences of MADS-box used for the construction of the tree are listed in the GenBank under the following accession numbers: *Mi*SEP3 (AEO45959), *Mi*SEP1 (ADX97328), AcSEP4 (ADU15479), MaSEP3MADS1 (ACJ64679), MaSEP3MADS2 (ACJ64678), MaSEP3MADS4 (ACJ64681), PpSEP3MADS5 (ABO27621), MdSEP4MADS4 (AAD51423), LeMADSRINSEP4 (NP_001234670), FvSEP2 (XP_004304204), CpMADS1 (ACD39982), VvMADS1 (XP_002283924), AtAGL3 (NP_849930), VlxVvAG (ABN46892), SlAGL1 (NP_001234187), AtAGL105 (NP_001119325), AtAGL14, (NP_192925), CsCAG2 (XP_004147393), VlxVvMADS1 (ABN46892), ZmAGAMOUS-like (NP_001105946), FvAGL62-like (XP_004305781), FvAGL15-like (XP_004305660), MtAGL6 (XP_003636892), MiAG2 (AER34989), PpAG (ABU41518). *Mi*, mango, *Mangifera indica; Ac*, kiwi fruit, *Actinidia chinensis*; *Ma*, banana, *Musa acuminata*; *Pp*, peach, *Prunus persica*; *Md*, apple, *Malus domestica*; *Le*, tomato, *Lycopersum esculentum*; *Cp*, papaya, *Carica papaya*; *At*, *Arabidopsis thaliana*; *Sl*, tomato, *Solanum lycopersicum*; *Cs*, cucumber, *Cucumis sativus*; *Vl × Vv*, grape, *Vitis labrusca × Vitis vinifera*; *Zm*, maize, *Zea mays*; *Fv*, woodland strawberry *Fragaria vesca*; *Mt*, *Medicago truncatula*; *Vv*, grape, *Vitis vinifera*

Therefore, phylogenetic analysis was performed using proteins from the SEP and AGL subfamilies from several fruits; the resultant dendrogram showed that MiMADS1 is more similar to AcSEP4 expressed in ripe kiwifruit (Fig. 3). Thus, MiMADS1 transcription factor could be involved in gene expression regulation during development and ripening of mango fruit. Several *MADS*-box genes were discovered in other fruits, and most of them are expressed in early stages of fruit development. In peach, an increase in expression was detected for two *MADS*-*box* genes during fruit development (Tadiello et al. 2009). Also, expression analysis of *MADS*-*box* transcripts in grapes was detected during fruit development (Elitzur et al. 2010). *MADS*-*box* genes in kiwifruit were required for floral meristem and floral organ specification (Varkonyi-Gasic et al. 2011). Due to the similarity between MADS1 form mango and other MICK-type proteins belonging to the SEP clade, we did a molecular modeling of MiMADS and docking with DNA. It is also known that SEP-like proteins interact with MADS by interaction with bridging proteins like TM5 (Leseberg et al. 2008). These studies in tomato suggest that two-hybrid approaches may help in identifying interactions during development and ripening in mango.

### MiMADS structural model and DNA interaction

**Fig. 4:**
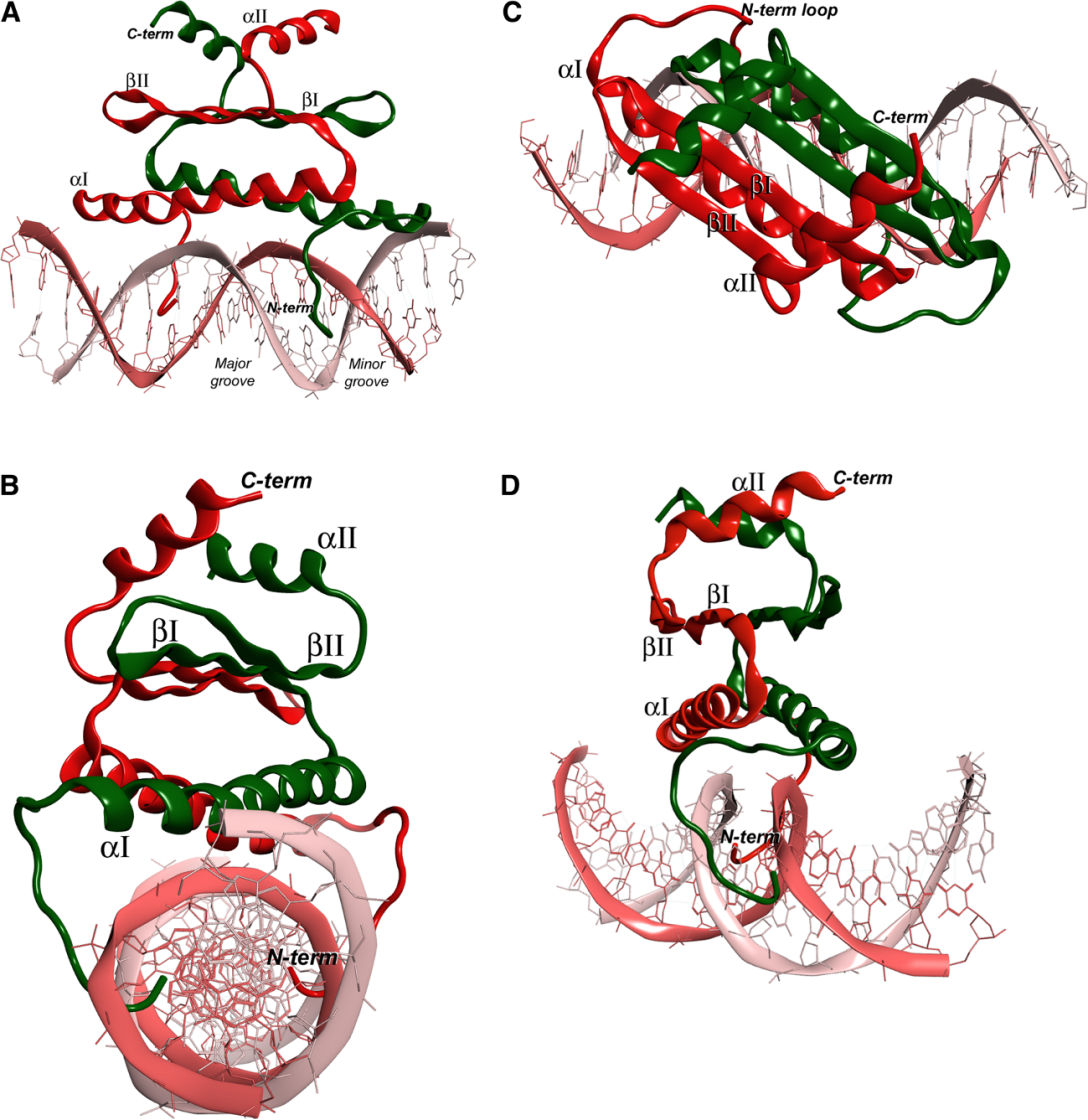
Ribbon representation of the DNA-binding domain of MiMADS1 bound to a double-stranded fragment. Each protein monomer is represented in *red* and *green* and helices and strands are numbered, and DNA on *pink*. **a** Side view of the complex. **b** Lateral view, where the N-terminus of MiMADS1 contacts the DNA **c** Top view, depicting the β-strand interaction between monomers. **d** Rotated lateral view showing the interaction of N-terminus with minor groove

The N-terminus of the amino acid sequence of MiMADS1 was readily modeled using MOE 2012.10, due to its identity to the crystal structure of MEF2A (Wu et al. 2010) (PDB 1EGW, 3KOV, 1C7U, 1TQP, 1N6J). The model obtained was a homodimer that also was docked to double strand DNA fragment (5′-TAAGCTAATAATAGCTT-3′) as reported for MEF2A (Fig. 4). The molecular model includes the first 72 residues of the MiMADS1 amino acids sequence, since the remainder of the molecule, probably involved in transcriptional activation, is novel and does not have significant identity with known structures. The amino acid identity with the template was 55 %, ensuring that the fold is conserved. Also, the conserved residues R3, V6, N16, K23, R24, K30, K31 and E34 are present and can establish specific contacts to DNA. The cognate fold serum-response factor (SRF) is comprised of a α − ββ − α dimer with an antiparallel sheet dimerization domain (Fig. 4).

The N-terminus was predicted as a loop, which has several residues that interacts with the DNA minor groove and the phosphate backbone. In a lateral view (Fig. 4a), the loops appear bound to the DNA, and in Fig. 4b the loops appear in the shape of a hook contacting the bottom of the minor groove with its N-term amine group. SRF-fold is different to other DNA-binding motifs such as the helix-loop-helix since in a small stretch of amino acids DNA is bound and dimerization is also achieved. A top view (Fig. 4c) shows the interaction between the antiparallel beta sheets, which are locked by one alpha helix from each monomer at 90° from the sheets orientation on top of them. The presence of the dimer has been reported by multi-angle light scattering (Wu et al. 2010) and by gel-filtration chromatography. A rotated lateral view (Fig. 4d) shows very clear the internal symmetry of the MiMADS dimer and the hooks interacting with the minor groove.

**Fig. 5:**
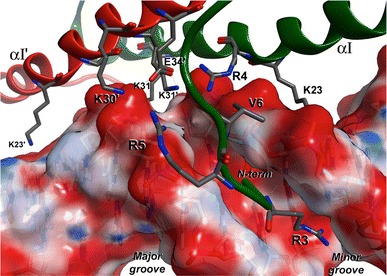
Details of the protein–DNA interactions predicted by molecular modeling. Arginines (R) and lysines (K) are critical for interaction with the phosphate DNA backbone. Prime numbering corresponds to the second monomer. *Red* color on the surface of DNA corresponds to negative charges of the phosphodiester bonds and *blue* corresponds to positive charges

It is known that MEF2A interacts with DNA mainly to the minor groove by contacts mediated from the N-terminal loop and helix αI (Fig. 5). The interactions between MiMADS1 and the two strands of DNA are mainly to the phosphate backbone, as found for the basic side chain of R5, R24, K30 and K31. The K31 amine side chain group established two H-bond contacts to phosphate groups at the two DNA strands, narrowing the minor groove cavity. The two K31 residues from the two monomers of MiMADS1 are adjacent from each other, possibly making a strong contribution to the DNA backbone stretching. E34 is other conserved residue found in MADS-box proteins. Its function is to coordinate an H-bond network through its carboxyl side chain that anchors K30, K31 and R24′ (from the other monomer) side chains in place, so it may be important for MiMADS1 dimerization and DNA backbone stretching. At the center of the helix αI is located the invariant G27, that it is important for preventing the steric hindrance at the close proximity to the DNA backbone (Fig. 5).

The conserved R3 side chain extends at the minor groove bottom and interacts to the nitrogen bases of DNA. The main interactions are through H-bonding to two adjacent pyrimidine rings at one strand of DNA and a ribose ring. At the same time, K23 contacts the purine rings from the complementary strand at the major groove. Both R3 and K23 are the main residues that contribute to binding specificity at the consensus sequence. The N-term G2 residue is another invariant residue that lie at the bottom of the minor groove and contacts to pyrimidine rings in the consensus sequence. V6 makes hydrophobic contacts to methylene carbons from the DNA backbone (Fig. 5). We should emphasize that this is a theoretical model and not an experimental crystal structure, but the model is consistent with the ionic charge interactions and this will wait to be demonstrated by the crystallization of MiMADS with a cognate DNA box.

### Expression of *MiMADS1* during mango development

**Fig. 6:**
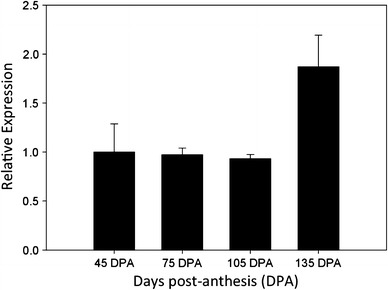
Expression of *MiMADS1* at different developmental stages of mango fruit (45, 75, 105 and 135 days post-anthesis). Fruits reached their physiological maturity at 135 DPA. *Bars* show the mean ± ES of three measurements (*n* = 3)

The expression pattern of *MiMADS*1 remained constant during development of mango and increased at mango physiological maturity (135 days post harvest, DPA) (Fig. 6). These results suggest a role of MiMADS1 for development of mango fruits and also a function during ripening. MiMADS1 could be up-regulating expression of genes that encode enzymes involved in ethylene biosynthesis based on the mango fruit from the developmental stage that showed higher expression levels (135 DPA). The rise in mRNA levels of *MiMADS1* at onset of ripening could be explained for auto regulation of its own expression. Fujisawa et al. (2011) showed that tomato MADS-RIN controlled its own expression and also they observed an increase of its expression at onset of ripening. *MADS*-*box* gene expression studies concerning development of fruits are scarce; nevertheless, there are reports of expression of *MADS*-*box* from banana mesocarp where it was found that *MADS*-*box* genes regulate ethylene synthesis (Liu et al. 2009). These results suggest that *MADS*-*box* genes have a function on fruit climatery and therefore in fruit ripening. However, it is necessary to test this hypothesis using tools like chromatin immunoprecipitation to confirm the regulation of ethylene pathway genes for MiMADS1. Also, it is important to evaluate the levels of the MiMADS protein since they are not necessarily correlated with the mRNA levels (Vogel and Marcotte 2012) and that specific post transcriptional or translational regulation exists during mango ripening.

 Until now, we found this cDNA encoding a MADS-box protein from mango fruit cv. “Keitt” and in the future it will be possible to pursue some of the above mentioned experiments. It is also possible to study the mRNA levels of *MiMADS1* during organ and flower development in mango in future studies, as initially described in *Arabidopsis*.

## Conclusion

We have identified a MADS-box transcription factor that could be important for mango fruit development, MiMADS1. Molecular modeling predicts that all-important residues are present for interactions in the minor groove of cognate double-stranded DNA. Future research on DNA-protein recognition and interaction will help to determine the MiMADS1 role within the transcriptional machinery acting during mango fruit development and ripening.
